# Reflecting on the association between video game addiction and early‐stage inhibitory control issues: Insights from Taiwan

**DOI:** 10.1111/adb.13404

**Published:** 2024-06-15

**Authors:** Lien‐Chung Wei, Kuen‐Hong Wu

**Affiliations:** ^1^ Department of Addiction Psychiatry, Taoyuan Psychiatric Center Republic of China Ministry of Health and Welfare Taoyuan City Taiwan

To the Editor of Addiction Biology,

We are writing to express our insights and reflections on the recently published study by Fathi et al.,^1^ ‘Video game addiction is associated with early stage of inhibitory control problems: An event‐related potential study using cued Go/NoGo task’, in your esteemed journal. This groundbreaking research offers valuable evidence linking video game addiction (VGA) to early‐stage inhibitory control issues, employing a cued Go/NoGo task to examine brain activity related to response inhibition.

Drawing from our experience and the context of similar research in Taiwan, we find this study's approach and findings both compelling and significant. For instance, a study on ‘Wisdom and the Net‐Entangled Generation: Discussing the Cooperation between Parents and Teachers in Addressing Adolescents' Mobile Internet Addiction’ in Taiwan^2^ also addresses the challenges posed by digital addiction among youth, suggesting the importance of collaborative efforts between parents and educators to mitigate the issue. These concerns echo the findings of Fathi et al., reinforcing the global relevance of understanding and addressing the cognitive impacts of digital addictions.

Furthermore, the study's use of event‐related potentials (ERPs) to explore the neural underpinnings of inhibitory control issues in individuals with VGA is remarkably insightful. It mirrors a broader academic interest in applying neuroscientific methods to study behavioural addictions. For example, the research by Chen^3^ on the integration of digital games in English vocabulary teaching hints at the nuanced interactions between digital media usage and cognitive processes, underscoring the need for balanced approaches in digital consumption.

It is also worth noting that Fathi et al.'s identification of specific ERP components (e.g., reduced N2 amplitude) provides a concrete neural basis for understanding the cognitive impairments associated with VGA. This aligns with trends in educational and psychological research that seek to identify the physiological and cognitive impacts of technology use among adolescents.^4^

While Fathi et al.'s study focuses on the early‐stage inhibitory control problems, it prompts further investigation into long‐term cognitive and social outcomes of VGA. It raises questions about the effectiveness of interventions and educational programs designed to mitigate these effects. The Taiwanese context, with its diverse approach to addressing digital and gaming addictions, offers a rich ground for comparative studies that could further elucidate the mechanisms and outcomes of such interventions.

In conclusion, Fathi et al.'s study is a significant contribution to the burgeoning field of research on digital addictions and their cognitive impacts. It underscores the urgency of developing informed, multi‐faceted strategies to address these issues. By integrating insights from diverse contexts, such as those provided by Taiwanese research, we can enhance our understanding and response to the challenges posed by digital addictions in our global society.

## CONFLICT OF INTEREST STATEMENT

The authors declare no conflicts of interest.

## FUNDING INFORMATION

This work did not receive funding from any sources.

## Data Availability

Data sharing is not applicable to this article as no new data were created or analysed in this study.
